# Periodontitis exacerbates pulmonary hypertension by promoting IFNγ^+^ T cell infiltration in mice

**DOI:** 10.1038/s41368-024-00291-2

**Published:** 2024-03-28

**Authors:** Xiaoqian Meng, Linjuan Du, Shuo Xu, Lujun Zhou, Boyan Chen, Yulin Li, Chumao Chen, Huilin Ye, Jun Zhang, Guocai Tian, Xuebing Bai, Ting Dong, Wenzhen Lin, Mengjun Sun, Kecong Zhou, Yan Liu, Wuchang Zhang, Shengzhong Duan

**Affiliations:** 1 Department of Endodontics, Shanghai Ninth People’s Hospital, College of Stomatology, Shanghai Jiao Tong University School of Medicine, Shanghai, China; 2 Laboratory of Oral Microbiota and Systemic Diseases, Shanghai Ninth People’s Hospital, College of Stomatology, Shanghai Jiao Tong University School of Medicine, National Center for Stomatology, National Clinical Research Center for Oral Diseases, Shanghai Key Laboratory of Stomatology, Shanghai, China; 3 Hongqiao International Institute of Medicine, Shanghai Tongren Hospital/Faculty of Basic Medicine, Key Laboratory of Cell Differentiation and Apoptosis of Chinese Ministry of Education, Shanghai Jiao Tong University School of Medicine, Shanghai, China; 4 Department of Orthodontics, Shanghai Ninth People’s Hospital, College of Stomatology, Shanghai Jiao Tong University School of Medicine, Shanghai, China; 5 Department of Periodontology, Shanghai Ninth People’s Hospital, College of Stomatology, Shanghai Jiao Tong University School of Medicine, Shanghai, China; 6 Stomatology Hospital, School of Stomatology, Zhejiang University School of Medicine, Hangzhou, China

**Keywords:** Periodontitis, Cardiovascular diseases

## Abstract

Uncovering the risk factors of pulmonary hypertension and its mechanisms is crucial for the prevention and treatment of the disease. In the current study, we showed that experimental periodontitis, which was established by ligation of molars followed by orally smearing subgingival plaques from patients with periodontitis, exacerbated hypoxia-induced pulmonary hypertension in mice. Mechanistically, periodontitis dysregulated the pulmonary microbiota by promoting ectopic colonization and enrichment of oral bacteria in the lungs, contributing to pulmonary infiltration of interferon gamma positive (IFNγ^+^) T cells and aggravating the progression of pulmonary hypertension. In addition, we identified *Prevotella zoogleoformans* as the critical periodontitis-associated bacterium driving the exacerbation of pulmonary hypertension by periodontitis, and the exacerbation was potently ameliorated by both cervical lymph node excision and IFNγ neutralizing antibodies. Our study suggests a proof of concept that the combined prevention and treatment of periodontitis and pulmonary hypertension are necessary.

## Introduction

Pulmonary hypertension (PH) remains a serious condition with a poor prognosis.^1^ PH is a progressive disease characterized by the gradual destruction of pulmonary arteries (PAs), elevated pulmonary arterial pressure, and hypertrophy and remodeling of the right ventricle (RV), culminating in RV failure and death.^2^ Common pathological features include remodeling of the distal PA and the infiltration of inflammatory cells.^3^ Limited treatment options for PH highlight the urgent need for more therapeutic targets.^4^

There is growing evidence suggesting that PH is a systemic disease influenced by the microbiota.^5–10^ Moreover, the role of the gut-lung axis in PH has garnered increasing attention.^5,7^ Numerous studies have explored the relationship between PH and the gastrointestinal tract, revealing a proinflammatory gut microbiota and altered microbial metabolites in individuals with PH.^11–14^ Furthermore, depletion of the gut microbiota through antibiotic intervention retards PH development in a rat model.^15^

On a different note, the significance of the oral-lung axis in respiratory diseases has gained prominence. Oral health status has been proposed to be a determinant of lung health because the microbes from the oral cavity can induce respiratory infections and inflammation after being inhaled into the lower respiratory tract.^16–18^ Additionally, associations have been observed between oral dysbiosis, notably periodontal disease, and various respiratory diseases.^17,19,20^ However, the specific role of the oral microbiota in maintaining lung homeostasis during PH remains largely unexplored.

Periodontitis serves as a paradigmatic illustration of oral microecological imbalance with epidemiological links to various systemic diseases, including respiratory infections.^21^ The interconnection between periodontitis and systemic comorbidities is often mediated through both local and systemic immune responses to oral pathogens.^22^ Moreover, substantial evidence indicates that inflammation plays a key role in human and experimental PH.^23^ Immune cell infiltration, which mainly involves T and B lymphocytes, macrophages, monocytes, mast cells and dendritic cells, is observed in remodeled pulmonary arteries in patients with PH.^24,25^ In addition, high levels of cytokines, chemokines and autoantibodies have been detected in animal models and patients with PH, suggesting local adaptive immune responses in the lung.^25,26^ Therefore, exploring the interaction between the oral microbiota and immunity in PH may offer novel therapeutic insights for managing PH.

Our primary goal in this study was to investigate the causal relationship between periodontitis and PH and to identify underlying mechanisms involved. First, we used a composite mouse model combining an experimental periodontitis model with hypoxia-induced PH to examine the impact of periodontitis on PH. Then, we conducted a comprehensive analysis of the oral and lung microbiota to identify the pivotal oral microbes involved in the influence of periodontitis on pathological progression of PH. Next, we explored the impacts of oral microbes on the immune response during PH. Finally, neutralizing antibodies were used to assess whether the immune response mediated the effect of oral microbes on exacerbating PH.

## Results

### Periodontitis exacerbates hypoxia-induced PH in mice

**Fig. 1:**
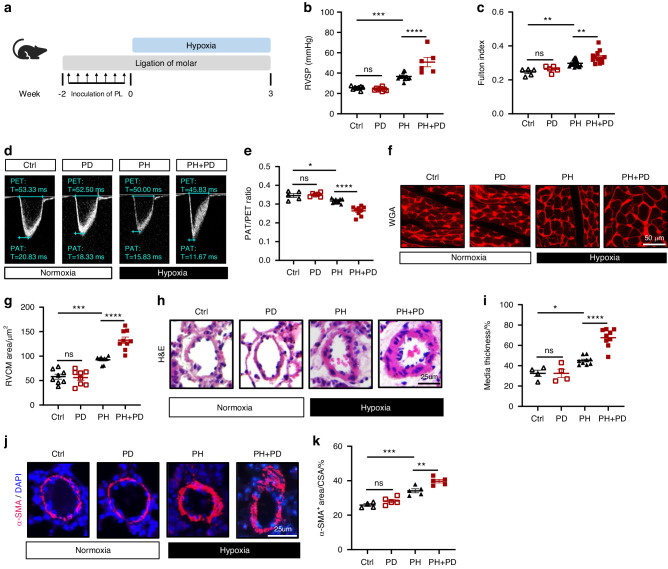
Periodontitis (PD) aggravates pulmonary hypertension (PH) in mice. **a** Schematic illustration of the experimental design. PD was induced by ligation of molar (at week -2) in combination with 7 times of inoculation of subgingival plaques (PL) taken from patients with periodontitis. Mice were exposed to normoxia (21% O_2_) or hypoxia (10% O_2_) for 3 weeks in a ventilated chamber. **b**, **c** Right ventricular systolic pressure (RVSP) **b** and Fulton index **c** determined by the ratio of right ventricle (RV) weight to left ventricle (LV) weight plus septum weight (RV/(LV + S)). *n* = 8:8:8:12 for RVSP, *n* = 5:5:16:13 for Fulton index. **d**, **e** Representative echocardiography images **d** and measurements of pulmonary artery (PA) function by PA acceleration time to ejection time (PAT/PET) ratio **e** after exposure to normoxia or hypoxia for 3 weeks. *n* = 5:5:11:9. **f** Representative wheat germ agglutinin (WGA) staining of RV sections. **g** Quantification of RV cardiomyocyte (RVCM) area based on WGA staining. *n* = 8:8:9:9. **h** Representative hematoxylin and eosin (H&E) staining of lung sections. **i** Quantification of medial thickness as a percentage of external diameter of PAs. *n* = 4:4:9:9. **j** Representative immunofluorescence staining of alpha-smooth muscle actin (α-SMA) in lung sections. **k** Quantification of α-SMA^+^ area as a percentage of cross-sectional area (CSA) of PAs. *n* = 4:5:5:5. Ctrl, nonligatured control. PD, ligature-induced periodontitis with oral infection of PL. PH, pulmonary hypertension. Data are presented as mean ± SEM. Two-way ANOVA **b**, **c**, **e**, **g**, **i**, **k** was used for statistical analysis. ns, not significant. *P < 0.05. **P < 0.01. ***P < 0.001. ****P < 0.000 1

To explore the impact of periodontitis on the progression of PH in mice, we established a mouse periodontitis model by ligating the bilateral maxillary second molars with silk suture and smearing subgingival plaques derived from periodontitis patients for 2 weeks (the composite model was referred as PD). The mice were then subjected to 3 weeks of hypoxia to induce PH (Fig. 1a). As expected, periodontitis resulted in substantial alveolar bone loss in mice under normoxic and hypoxic conditions (Fig. S1a and S1b). Increased RV afterload is a result of elevated pulmonary circulation pressure, which eventually causes RV hypertrophy and dysfunction.^27^ Mice in the PH + PD group exhibited significant increases in right ventricular systolic pressure (RVSP) and Fulton index, two markers of RV hypertrophy, compared with mice in the PH group (Fig. 1b, c). However, periodontitis did not affect RVSP or Fulton index in mice without PH (Fig. 1b, c). A decrease in the pulmonary artery acceleration to ejection time (PAT/PET) ratio often indicates compromised pulmonary vascular function in PH mice. Echocardiography demonstrated a smaller PAT/PET ratio in PH + PD mice than in PH mice, but no difference was observed between PD mice and Ctrl mice (Fig. 1d, e). To evaluate cardiomyocyte hypertrophy of right ventricle, we carried out wheat germ agglutinin (WGA) staining of mouse heart sections. The results demonstrated a larger cardiomyocyte size in PH + PD mice compared with PH mice (Fig. 1f, g). In addition, hematoxylin and eosin (H&E) staining (Fig. 1h, i) and immunofluorescence staining of lung sections (Fig. 1j, k) respectively revealed significantly thickened medial walls and increased α-smooth muscle actin positive (α-SMA^+^) areas in distal PAs of PH + PD mice compared with PH mice, suggesting more severe vascular remodeling of pulmonary artery in PH + PD mice than that in PH mice. Collectively, our data showed that periodontitis exacerbated the progression of hypoxia-induced PH.

### Periodontitis increases the accumulation of *Prevotella zoogleoformans* (*P. zoogleoformans*) in the oral cavity and lungs of mice with PH

**Fig. 2:**
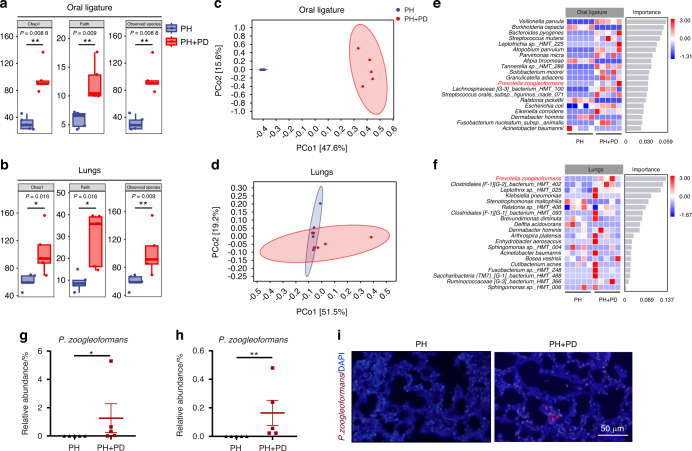
PD alters both oral and lung microbiota in hypoxia-induced PH mice. **a**, **b** Chao1, Faith’s phylogenetic diversity (Faith’s) and Observed species of microbiota in oral ligature **a** and lungs **b**. *n* = 5 per group. **c**, **d** Principal coordinate analysis (PCoA) of microbiota in oral ligature **c** and lungs **d**. *n* = 5 per group. **e**, **f** Random Forest regression analysis of oral **e** and lung **f** microbiota. Identified species were ranked by importance and relative abundance of the indicated species in oral ligatures and lungs. *n* = 5 per group. **g**, **h** Relative abundance of *Prevotella zoogleoformans* (*P. zoogleoformans*) in oral ligatures **g** and lungs **h**. *n* = 5 per group. **i** Fluorescence in situ hybridization for *P. zoogleoformans* in lung sections. Data are presented as mean ± SEM. Student’s *t* test **g**, **h** was used for statistical analysis. *P < 0.05. **P < 0.01

Next, we utilized full-length 16 S rRNA gene sequencing to examine bacterial changes in the oral cavity and lungs of the mice. α-diversity analysis revealed increases in Chao1 richness, Faith phylogenetic diversity, and Observed species richness in oral ligatures and the lungs of PH + PD mice versus PH mice (Fig. 2a, b). Additionally, differences in bacterial composition between PH and PH + PD animals were observed by principal coordinate analysis based on Bray‒Curtis distances (Fig. 2c, d). Overall, the structural differences in bacterial communities were significant, categorizing the samples into distinct groups (PH and PH + PD). These data demonstrated that periodontitis markedly remodeled the bacterial ecology in the oral cavity and pulmonary niche in mice.

Moreover, microbial differences at the levels ranging from phylum to species were analyzed to determine whether there were bacterial taxa that were changed in a similar trend in the lungs and oral cavity in the presence of periodontitis. Intriguingly, *P. zoogleoformans*, the bacterial species that ranked the highest in the lungs, was also enriched in the oral cavity of PH + PD mice compared with PH mice, according to the random forest regression analysis (Fig. 2e, f). The Linear discriminant analysis effect size (LEfSe) consistently showed that 14 bacterial species including *P. zoogleoformans* were enriched in the oral cavity of the PH + PD group, while 5 bacterial species were enriched in the PH group (Fig. S2a and S2b). As shown by the relative abundance of species-level microbiota, *P. zoogleoformans* was significantly enriched in both oral cavity and lungs in the PH + PD group versus PH group (Fig. 2g, h, S2c and S2d). Furthermore, *P. zoogleoformans* was observed in the lung sections of PH + PD mice, but not in those of PH mice, indicating that it ectopically colonized in lungs after periodontitis induction (Fig. 2i). These findings altogether demonstrated that periodontitis promoted the dysregulation of the oral and lung microbiota and the accumulation of *P. zoogleoformans* in the lungs of PH mice.

### *P. zoogleoformans* promotes the progression of PH in mice

**Fig. 3:**
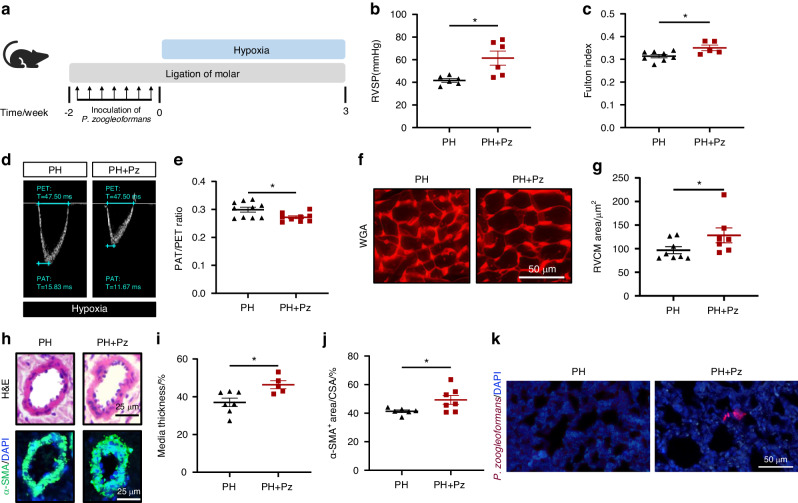
*P. zoogleoformans* exacerbates PH in mice. **a** Schematic illustration of the experimental design. **b**, **c** RVSP **b** and Fulton index **c** determined by the ratio of (RV/(LV + S)). *n* = 6:6 for RVSP, *n* = 8:5 for Fulton index. **d**, **e** Representative echocardiography images **d** and measurements of PA function by PAT/PET ratio **e** after exposure to hypoxia for 3 weeks. *n* = 10:9. **f** Representative WGA staining of RV sections. **g** Quantification of RVCM area based on WGA staining. *n* = 8:7. **h** Representative H&E staining and immunofluorescence staining of α-SMA of lung sections. **i** Quantification of medial thickness as a percentage of external diameter of PAs. *n* = 7:5. **j** Quantification of α-SMA^+^ area as a percentage of cross-sectional area (CSA) of PAs. *n* = 6:7. **k** Fluorescence in situ hybridization for *P. zoogleoformans* in lung sections. Pz, ligature-induced periodontitis with oral infection of *P. zoogleoformans*. Data are presented as mean ± SEM. Student’s *t* test (**b**, **c**, **e**, **g**, **i**, **j**) was used for statistical analysis. *P < 0.05

To examine the contribution of *P. zoogleoformans* to the progression of PH, mice were either orally inoculated with *P. zoogleoformans* for 2 weeks or not, and then subjected to 3 weeks of hypoxic treatment (Fig. 3a). The combined treatment of *P. zoogleoformans* inoculation and ligation of molars (Pz) contributed to significant alveolar bone resorption, suggesting the successful modeling of periodontitis (Fig. S3a, S3b and S3c). RVSP and Fulton index were all significantly increased in PH + Pz mice versus PH mice, indicating that *P. zoogleoformans* worsened PH and right ventricular dysfunction in mice (Fig. 3b, c). Instead, *Atopobium parvulum* and *Streptococcus mutans* did not affect these parameters (Fig. S4a, S4b and S4c), suggesting the specific role of *P. zoogleoformans* in promoting PH. Echocardiography revealed a decrease in the PAT/PET ratio in PH + Pz mice (Fig. 3d, e). Consistently, PH + Pz mice exhibited a more pronounced increase in cardiomyocyte size and RV hypertrophy than PH mice (Fig. 3f, g). The histopathological examinations confirmed severe PH-associated vascular remodeling in PH + Pz mouse lungs, as evidenced by increases in wall thickness and α-SMA-positive areas of pulmonary arteries (Fig. 3h–j). Additionally, the results of fluorescence in situ hybridization demonstrated the presence of *P. zoogleoformans* in the lungs of PH + Pz mice, but not in those of PH mice (Fig. 3k). Put all these data together, it was indicated that oral inoculation of *P. zoogleoformans* potently aggravated hypoxia-induced PH.

### *P. zoogleoformans* elevates the accumulation of interferon gamma (IFNγ)-producing T cells

**Fig. 4:**
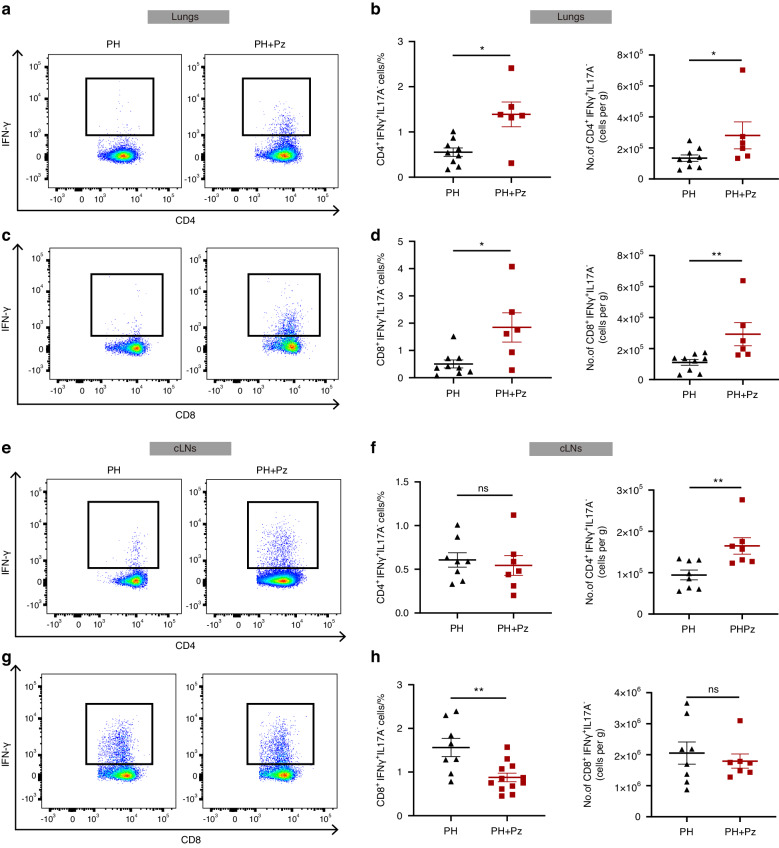
*P. zoogleoformans* promotes the accumulation of IFNγ^+^ T cells in mouse lungs. Periodontitis was performed 14 days before the induction of PH. Samples were analyzed 7 days after PH induction. **a**, **b** Representative flow cytometry plots of CD4^+^IFNγ^+^ T cells **a** and quantification of the percentage of CD45^+^ cells and number of CD4^+^IFNγ^+^ T cells **b** in lungs. *n* = 9:6. **c**, **d** Representative flow cytometry plots of CD8^+^IFNγ^+^ T cells **c** and quantification of the percentage of CD45^+^ cells and number of CD8^+^IFNγ^+^ T cells **d** in lungs. *n* = 9:6. **e**, **f** Representative flow cytometry plots of CD4^+^IFNγ^+^ T cells **e** and quantification of the percentage of CD45^+^ cells and number of CD4^+^IFNγ^+^ T cells **f** in cervical lymph nodes (cLNs). *n* = 8:7. **g**, **h** Representative flow cytometry plots of CD8^+^IFNγ^+^ T cells **g** and quantification of the percentage of CD45^+^ cells and number of CD8^+^IFNγ^+^ T cells **h** in cLNs. *n* = 8:7. Data are presented as mean ± SEM. Student’s *t* test **b**, **d**, **f**, **h** was used for statistical analysis. ns not significant. *P < 0.05. **P < 0.01

Dysregulated immune response is a prominent pathological hallmark of PH, which is characterized by the overwhelming infiltration of immune cells including T lymphocytes into the pulmonary vasculature.^28,29^ To identify the underlying mechanisms by which *P. zoogleoformans* exacerbates PH, we focused on profiling immune cells in the lungs and cervical lymph nodes (cLNs) of both PH and PH + Pz mice 7 days after the hypoxia treatment using the flow cytometry. There was no significant change in the numbers of total CD4^+^ or CD8^+^ T cells between the PH + Pz and PH groups (Fig. S5a and S5b). However, the percentages and numbers of CD4^+^ IFNγ^+^ T cells and CD8^+^ IFNγ^+^ T cells, but not that of interleukin-17A positive (IL17A^+^) T cells, were notably increased in the lungs in the PH + Pz groups versus PH groups (Fig. 4a–d, S5c and S5d). In addition, oral inoculation of *P. zoogleoformans* significantly increased the numbers of CD4^+^ IFNγ^+^ T cells in cLNs, while their proportion remained unchanged (Fig. 4e, f). The numbers of CD8^+^ IFNγ^+^ T cells were comparable in cLNs between the PH and PH + Pz groups, indicating that the decrease of relative proportion of CD8^+^ IFNγ^+^ T cells could be due to the increases of other types of immune cells (Fig. 4g, h). These results together showed that IFNγ^+^ T cells could mediate *P. zoogleoformans*-induced aggravation of PH.

### Cervical lymph nodes are crucial for the exacerbation of PH caused by *P. zoogleoformans*

**Fig. 5:**
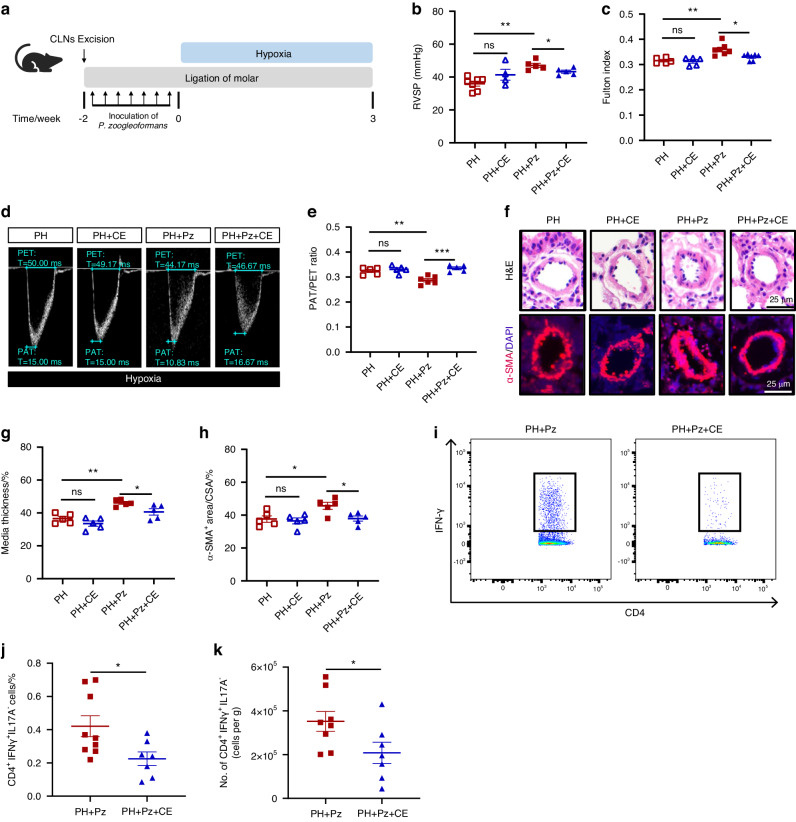
Surgical excision of cLNs alleviates the exacerbating effects of *P. zoogleoformans* on PH. **a** Schematic illustration of the experimental design. **b**, **c** RVSP **b** and **c** Fulton index determined by the ratio of (RV/(LV + S)). *n* = 5: 5:7:7 for RVSP, *n* = 7: 4:5:5 for Fulton index. **d**, **e** Representative echocardiography images **d** and measurements of PA function by PAT/PET ratio **e** after exposure to hypoxia for 3 weeks. *n* = 5: 5:6:5. **f** Representative H&E staining and immunofluorescence staining of α-SMA of lung sections. **g** Quantification of medial thickness as a percentage of external diameter of PAs. n = 5: 5:5:5. **h** Quantification of α-SMA^+^ area as a percentage of cross-sectional area (CSA) of PAs. *n* = 5: 5:5:5. **i** Representative flow cytometry plots of CD4^+^IFNγ^+^ T cells in lungs. **j**, **k** Quantification of the percentage of CD45^+^ cells and number of CD4^+^IFNγ^+^ T cells in lungs. *n* = 8:7. CE, cLN excision. Data are presented as mean ± SEM. Two-way ANOVA **b**, **c**, **e**, **g**, **h** and Student’s *t* test **j**, **k** were used for statistical analysis. ns not significant. *P < 0.05. **P < 0.01. ***P < 0.001

cLNs play a crucial role in initiating immune responses to pathogenic antigens in the oral cavity. To elucidate their role in *P. zoogleoformans*-induced exacerbation of PH, cLN excision (CE) was performed in mice followed by ligation of molars and inoculation of *P. zoogleoformans* as well as induction of hypoxia (Fig. 5a). CE significantly inhibited the increases in RVSP and Fulton index induced by *P. zoogleoformans*, but had no effect on PH mice (Fig. 5b, c). Moreover, the decrease in PAT/PET ratio was reversed by cLN excision (Fig. 5d, and e). The size of cardiomyocytes in the PH + Pz group, but not PH group, was also markedly decreased by CE, suggesting the amelioration of *P. zoogleoformans*-enhanced right ventricle hypertrophy (Fig. S6a and S6b). Consistently, pulmonary artery remodeling was significantly alleviated after the excision of cLNs, as shown by the decreased medial thickness and smaller α-SMA^+^ area in the PH + Pz + CE group versus PH + Pz group (Fig. 5f–h). However, CE failed to alleviate pulmonary artery remodeling in PH mice (Fig. 5f–h). We next evaluated the effects of CE on the infiltration of IFNγ^+^ T cells in lungs of PH + Pz mice by flow cytometry. The removal of cLNs significantly suppressed CD4^+^ IFNγ^+^ T cell accumulation in the lungs, while it did not affect the numbers of CD8^+^ IFNγ^+^ T cells (Fig. 4a–d, 5i–k, S6c and S6d), indicating that the enriched CD4^+^ IFNγ^+^ T cells in lungs come from cLNs in PH + Pz mice. These results cumulatively demonstrated that cLNs were indispensable for *P. zoogleoformans*-induced exacerbation of PH and its associated cardiac and vascular remodeling.

### IFNγ neutralization mitigates PH exacerbated by *P. zoogleoformans*

**Fig. 6:**
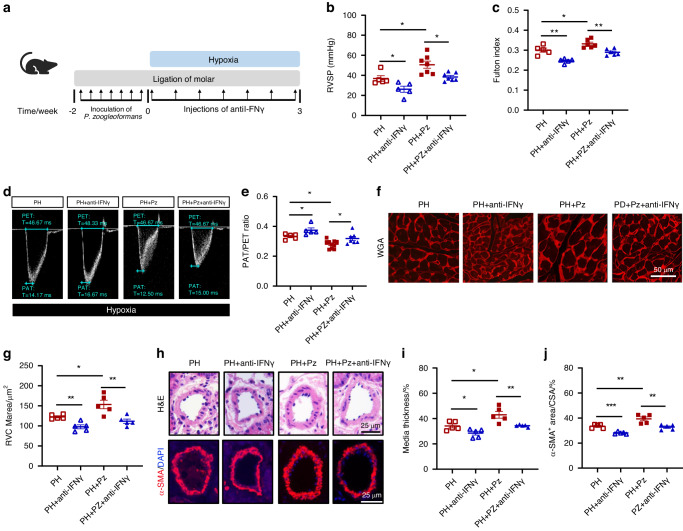
IFNγ neutralization constrains P*. zoogleoformans*-induced exacerbation of PH. **a** Schematic illustration of the experimental design. **b**, **c** RVSP **b** and **c** Fulton index determined by the ratio of (RV/(LV + S)). *n* = 5: 5:7:7 for RVSP, *n* = 5: 5:6:6 for Fulton index. **d**, **e** Representative echocardiography images **d** and measurements of PA function by PAT/PET ratio **e** after exposure to hypoxia for 3 weeks. *n* = 5: 5:10:7. **f** Representative WGA staining of RV sections. **g** Quantification of RVCM area based on WGA staining. *n* = 5:5:5:5. **h** Representative H&E staining and immunofluorescence staining of α-SMA of lung sections. **i** Quantification of medial thickness as a percentage of external diameter of PAs. *n* = 5:5:5:5. **j** Quantification of α-SMA^+^ area as a percentage of cross-sectional area (CSA) of PAs. *n* = 5:5:5:5. Anti-IFNγ, IFNγ-neutralizing antibodies treatment. Data are presented as mean ± SEM. Two-way ANOVA **b**, **c**, **e**, **g**, **i**, **j** was used for statistical analysis. *P < 0.05. **P < 0.01. ***P < 0.001

We next determined whether IFNγ induced by *P. zoogleoformans* was involved in the exacerbation of PH. For this purpose, mice were intraperitoneally injected with IFNγ neutralizing antibodies every three days during hypoxia exposure (Fig. 6a). Neutralization of IFNγ significantly decreased RVSP and the Fulton index of PH mice and PH + Pz mice (Fig. 6b, c). In addition, echocardiography demonstrated that neutralization of IFNγ improved PAT/PET ratio in PH mice and reversed the decrease of PAT/PET ratio induced by *P. zoogleoformans* (Fig. 6d, e). Similarly, the cardiomyocyte hypertrophy of RV was significantly reduced after IFNγ neutralization in the PH group and PH + Pz group, as shown by the WGA staining results (Fig. 6f, g). Moreover, blocking IFNγ ameliorated pulmonary vessel wall remodeling in PH mice and reversed the *P. zoogleoformans-*induced aggravation in vascular remodeling of pulmonary arteries, as shown by H&E and α-SMA staining (Fig. 6h–j). Thus, neutralization of IFNγ markedly reversed the effects of *P. zoogleoformans* on hypoxia-induced PH, emphasizing the crucial role of IFNγ.

## Discussion

**Fig. 7:**
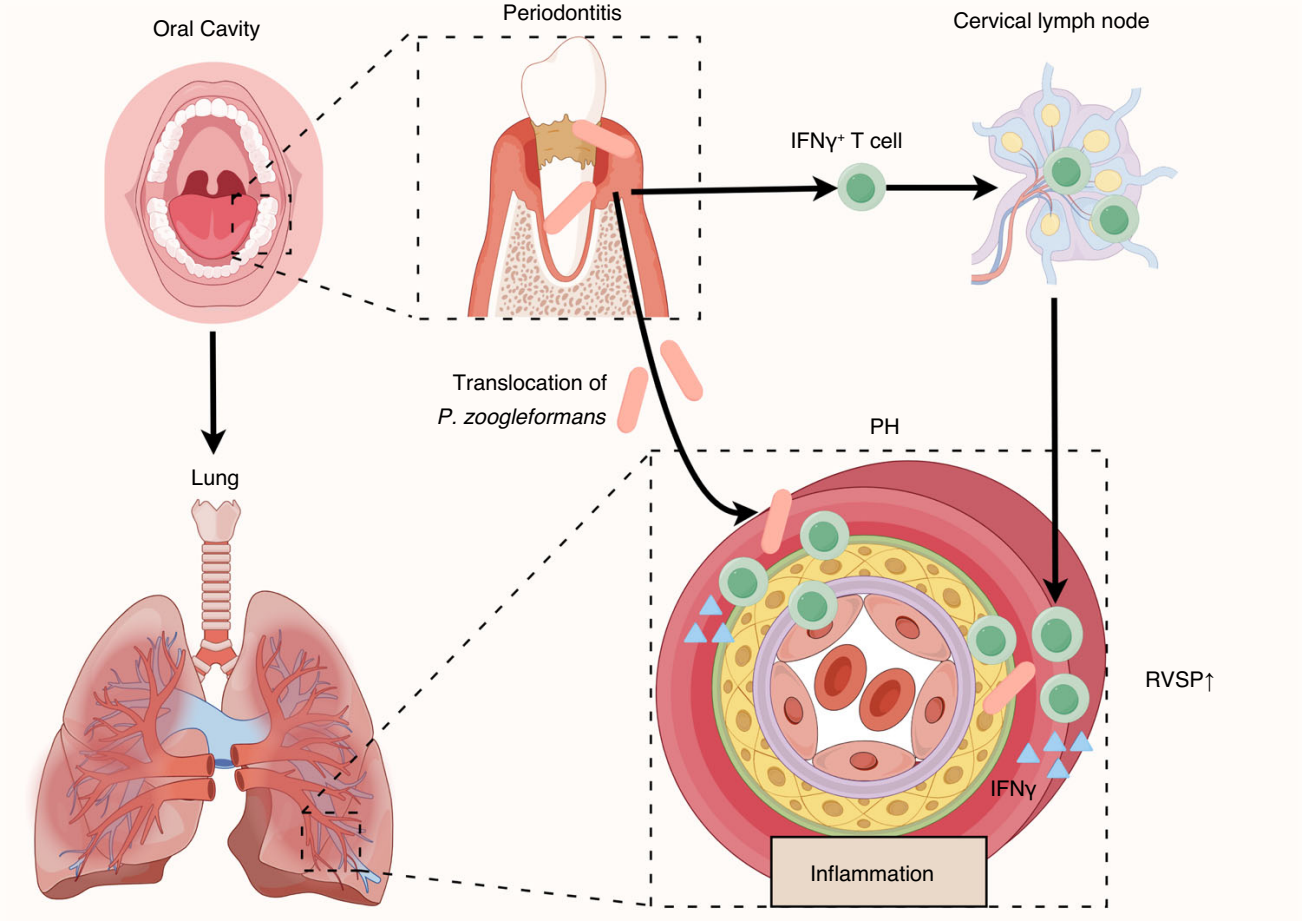
Working model illustrating the mechanisms that mediate the aggravation of pulmonary hypertension (PH) by periodontitis. Periodontitis aggravates PH through an oral-lung axis. The specific mechanisms include: (1) oral pathogen *P. zoogleoformans* induces the expansion of IFNγ^+^ T cells in cervical lymph nodes (cLNs), and (2) periodontitis facilitates the translocation of *P. zoogleoformans* to the lungs, leading to increased infiltration of IFNγ^+^ T cells in the lung. RVSP, right ventricular systolic pressure

Multiple studies have shown that the oral microbiota could shape the pulmonary microbiota by invading the lungs, leading to lung dysfunction and an increase in proinflammatory cytokines, which are correlated with the progression of PH.^18,24,30^ Despite these observed associations, there is a notable lack of definite evidence linking the oral microbiota to the worsening of PH. In this study, a composite mouse model incorporating periodontitis and PH was used, and we showed for the first time that periodontitis exacerbated the progression of PH. Mechanistically, periodontitis fostered ectopic colonization of *P. zoogleoformans* in the lungs, upregulating the infiltration of IFNγ^+^ T cells and ultimately intensifying the severity of PH (Fig. 7).

Our data have clearly demonstrated that periodontitis exacerbates the progression of PH. In the current study, we focused on the role of periodontal disease, which is a paradigmatic manifestation of oral microbial dysbiosis, in the pathogenesis of PH. We observed that periodontitis promoted RV dysfunction and aggravated pulmonary arterial remodeling in hypoxia-induced PH. PH is a vasculopathy characterized by excessive pulmonary vasoconstriction and aberrant vascular remodeling processes, which involve uncontrolled proliferation of endothelial and smooth muscle cells, as well as fibroblasts.^3,24^ Interestingly, evidence from epidemiological studies suggests that individuals with periodontitis exhibit notable endothelial dysfunction, while periodontal treatment increases surrogate measures of endothelial function, such as flow-mediated dilation of the brachial artery.^31,32^ Additionally, numerous clinical studies have revealed that hypertensive individuals with periodontitis exhibit elevated blood pressure, and intensive periodontal treatment significantly reduces blood pressure.^33,34^ Although it has been reported that periodontitis adversely affects vascular function, there remains insufficient evidence clarifying the role of periodontitis in PH.^35^ Our study demonstrated that periodontitis significantly exacerbated PH, suggesting a direct connection between periodontitis and PH. Consequently, appropriate oral care, such as periodontal treatment, is a potentially effective strategy for addressing the comorbidity of periodontal disease and PH.

Periodontitis further worsens lung microbiota dysbiosis caused by PH through the oral-lung axis, potentially contributing to the further aggravation of PH. Accumulating evidence suggests that alterations in the airway microbiota have vital effects on the progression of PH.^7^ Our findings showed that periodontitis not only increased microbial diversity but also altered the composition of the lung microbiota during PH. Consistent with a previous study, Ralstonia, which was as a major genus in oropharynx respiratory tract microorganism samples of PH patients, was also the predominant genus in lung samples of the PH and PH + PD groups in our hypoxia-induced mouse model, indicating that the hypoxia-induced mouse model could mimic the changes in the pulmonary microbiota of patients with PH to some extent.^10^ More importantly, periodontitis promoted the enrichment of oral resident bacteria in the lungs, including *P. zoogleoformans*. These findings suggested that periodontitis may further exacerbate the preexisting microecological disorders associated with PH by facilitating the translocation of oral bacteria to the lungs. Correspondingly, previous findings revealed that periodontitis could not only induce oral microbiota dysbiosis but also affect distal organs/tissues, including atherosclerotic plaques, placenta, and lungs.^35^ Periodontitis has also been linked to alterations in the gut microbiota in humans and mice.^36,37^ While the oral microbiome is recognized as a contributing factor in various cardiovascular diseases, its connection to PH has received limited attention. Therefore, our data strengthen the evidence of microbial communication along the oral-lung axis, emphasizing its significance in the pathogenesis of PH in the context of poor periodontal health.

Periodontitis with oral inoculation of *P. zoogleoformans* contributes to the progression of hypoxia-induced PH. Through analysis of microbiota in the oral cavity and lungs, we identified the gram-negative bacterium *P. zoogleoformans* as a potential key contributor to the exacerbation of PH. Further experiments demonstrated that oral inoculation of *P. zoogleoformans* potentiated the response to chronic hypoxia in mice. These results highlighted the importance of the oral bacterium *P. zoogleoformans*, which likely worked through ectopic colonization in the lungs, in the exacerbation of PH by periodontitis. In fact, previous findings suggest that periodontal pathobionts can translocate via the oro-pharyngeal route, ectopically colonizing the lungs.^22,38^ Moreover, ectopically colonized periodontal pathogens from the oral cavity may modulate immune responses outside the oral cavity, contributing to systemic disease development.^35,39–41^ Similarly, a previous study has shown that airway delivery of *S. salivarius* induces experimental PH in rats, indicating the connections between the oral microbiota and PH.^42^ The combined findings of our study and previous research highlight that *P. zoogleoformans* can be remotely disseminated and trigger immune responses that actively contribute to its pathogenic potential. Considering the potential pathogenic role of *P. zoogleoformans* in PH, targeting *P. zoogleoformans* and preventing ectopic pulmonary colonization are promising strategies for the treatment of PH as a comorbidity of periodontitis.

*P. zoogleoformans* predominantly mobilized IFNγ^+^ T cells during PH. In the current study, we found that oral inoculation of *P. zoogleoformans* facilitated the infiltration of IFNγ^+^ T cells in the cLNs and lungs. Removal of cLNs abrogated the *P. zoogleoformans*-induced increase in the pulmonary infiltration of IFNγ^+^ T cells, especially CD4^+^ IFNγ^+^ T cells, indicating that CD4^+^ IFNγ^+^ T cells may migrate to the lungs from cLNs. Finally, we demonstrated that blocking IFNγ effectively limited the exacerbation of PH in mice after inoculation with *P. zoogleoformans*, suggesting that *P. zoogleoformans* aggravated PH in an IFNγ-dependent manner. Previous studies have shown that knockout of IFNγ in mice reverses PH caused by *Pneumocystis* infection,^43^ indicating the crucial role of this cytokine in the development of PH. Furthermore, increased IFNγ levels in the plasma and lungs have been observed in patients with various types of PH, and a rare but extremely serious adverse effect of clinical interferon treatment is PH.^41,44–48^ In light of these findings and our results, it can be inferred that blocking IFNγ is beneficial for treating PH.

Collectively, our results demonstrated a direct relationship between periodontitis and PH. Mechanistically, oral bacterium *P*. zoogleoformans promoted pulmonary inflammation, subsequently contributing to the pathogenesis of PH via IFNγ. These findings suggest that targeting periodontitis or *P. zoogleoformans* holds promise for refining the precision of PH treatment, and that IFNγ is a promising therapeutic target of PH especially as a comorbidity of periodontitis.

## Materials and methods

### Mice

Eight-week-old male C57BL/6 mice (weight 23–25 g) were obtained from Beijing Vital River Laboratories (Beijing, China) and housed in a standard environment (23 °C; 12/12-h light/dark cycle) with ad libitum access to water and food at an SPF facility. To establish a consistent commensal microbiota, mice from different cages were cross-housed on soiled bedding for 2 weeks before the experiments. Ethics approval for all animal studies was granted by the Institute of Animal Care and Use Committee of Cyagen (Approval No. ACU21-A041; Cyagen Biosciences Inc.) and adhered to ARRIVE guidelines.

### Animal experiments

Mice were randomly assigned to different groups, and then exposed to hypoxia (10% O_2_) for 3 weeks to establish PH or room air as a control. In IFNγ neutralization experiment, mice received intraperitoneal injections of 200 μg anti-IFNγ antibodies (BP0055, BioXcell) in 200 μL PBS every 3 days (seven doses in total) as previously described.^49^ Heart and lung samples were subjected to perfusion, fixation, and embedding for further analysis. The right lung was snap-frozen in nitrogen (N_2_) and stored at −80 ^°^C.

Periodontitis was induced by bilateral ligation around the maxillary second molars.^50,51^ Subgingival plaque (PL) samples collected from patients with moderate to severe chronic periodontitis were centrifuged and resuspended in sterile 2% carboxymethylcellulose, and then orally inoculated (100 μL per mouse) once every two days for 2 weeks.^52^

For the Pz mouse model, periodontitis was induced by bilateral ligation around the maxillary second molars, and a total of 1 × 10^9^|colony-forming units (CFU) of *P. zoogleoformans* in sterile 2% carboxymethylcellulose were orally inoculated (100 μL per mouse), once every two days for 2 weeks.

Mice in the control group and PH group were subjected to sham ligation and treated with 2% carboxymethylcellulose without bacteria. Oral ligatures were maintained throughout the experiments for the periodontal ligation groups or for 3 h before the end of experiments in the periodontal sham ligation groups.^37^ After the mice were sacrificed, the ligatures were collected and stored at -80 °C for subsequent microbiome analysis. Mouse maxillae were prepared for analysis after the removal of soft tissues, overnight bleaching with 3% hydrogen peroxide solution, and staining with 1% methylene blue aqueous solution.^53^

For the cLN excision surgery, a small incision was made on the skin overlying the salivary glands (the base of the left and right ear junctions) and the skin was separated from the underlying fascia. With the aid of a surgical microscope, cLNs around the salivary glands were removed.^54^

### Echocardiographic assessment

Mice were anesthetized with 1.5% isoflurane and subjected to echocardiography with a 30 MHz probe (Vevo 3100LT, Visual Sonics, Ontario, Canada). Body temperature was maintained at 37 °C and heart rate was maintained at 500–550 beats per min. Pulse-wave Doppler echocardiography was used to record pulmonary blood outflow at the level of the aortic valve in short-axis view to measure PAT and PET. The sample volume was placed in the center of the color Doppler PA.^55^ Assessments including PAT and PET were performed using Visual Sonics Vevo 3100 analysis software (v.3.2.6) and averaged over 5 cardiac cycles.^56^

### Hemodynamic and ventricular weight measurements

Mouse RVSP was monitored using radiotelemetry according to established methods.^57,58^ Mice were anesthetized and hearts were exposed between the fourth and fifth ribs with two blunt forceps. Pressure transmitter (HDX10, Data Sciences International) was inserted into the RV through a xiphocostal angle approach.^59^ Pressure was recorded every 10 s for 3–5 min. The RV was carefully isolated from the left ventricle (LV) using curved tenotomy scissors, and the septum (S) with the LV was retained. RV and (LV + S) were weighed, and the Fulton index was calculated as RV/(LV + S).

### Histological analysis

Lungs and hearts were fixed with 4% paraformaldehyde for 24 h and embedded in paraffin. Sections (5 μm thick) were subjected to H&E staining. For immunofluorescence staining, antigen retrieval was performed by boiling the samples in citric acid buffer (pH 6.0) for 30 min. The sections were blocked in 5% goat serum for 60 min and incubated with primary antibodies (BM0002, Boster Biotech) at 4 °C overnight. This was followed by incubation with fluorochrome-conjugated secondary antibodies (Thermo Fisher Scientific) at room temperature for 2 h and DAPI (P36931, Thermo Fisher Scientific) counterstaining. WGA staining was performed at 37 °C in the dark for 2 h. Wall thickness in experimental PH models was assessed using vessels (<100 μm in diameter) and bronchial arteries were excluded. Wall thickness was calculated with the formula ((2×medial wall thickness/external diameter) ×100).^59^ The sections were inspected using a fluorescence microscope (Leica).

### Fluorescent in situ hybridization

Formalin-fixed, paraffin-embedded lung tissues were de-waxed and sequentially immersed in ethanol. The sections were washed with sterile PBS and incubated with lysozyme buffer (7 mg/mL) at 37 °C for 20 min. After being pretreated with hybridization buffer (0.9 NaCl, 20 mmol/L Tris-Cl (pH 8), and 0.1% (wt/vol) SDS) at 48 °C for 20 min, the sections were incubated with hybridization buffer containing 25% formamide and 0.2 μmol/L Alexa Fluor 594-labeled *P. zoogleoformans* probe (5′-TCCTTTACGGTTACGCACTTC-3′) in a dark humid chamber at 48 °C for 2 h. The sections were then washed with hybridization washing solution (0.02 mol/L NaCl, 20 mmol/L Tris-Cl (pH 8), 0.5 mol//L EDTA and 0.1% (wt/vol) SDS) for 25 min at 48 °C.^60,61^ The sections were counterstained with DAPI (P36931, Thermo Fisher Scientific).

### Bacterial growth

*P. zoogleoformans* (CCUG 20495 T) was cultured in chopped meat carbohydrate broth (HLM0052, Haling Biological Technology Co., Ltd) supplemented with hemin (0.5 mg) and vitamin K1 (5 mg) per 100 mL of broth. *A. parvulum* (CCUG 69363 A) was cultured on chocolate agar (HLM1802B, Haling Biological Technology Co., Ltd). *S. mutans* (UA159) was cultured in brain heart infusion (237500, BD Pharmingen) broth. All bacteria were grown anaerobically at 37 °C for 48 h. Bacterial DNA was extracted from a single colony, and the identity of individual isolates was verified by nearly full-length 16 S rRNA gene sequencing.

### Microbiome analysis

DNA extraction from the oral ligatures and lungs was performed using OMEGA Soil DNA Kits (M5635-02, Omega). The bacterial 16 S rRNA gene was amplified by PCR using primers (27 F: 5′-AGAGTTTGATCCTGGCTCAG-3′, 1492 R: 5′-GGTTACCTTGTTACGACTT-3′) with sample-specific barcodes. PCR amplicons were purified, quantified, and sequenced. Microbiome bioinformatics was performed by QIIME software. The raw data have been submitted to the NCBI Short Read Archive under the BioProject accession number PRJNA1042941.

### Immune cell analysis

For flow cytometry, single-cell suspensions of mouse cLNs and lungs were prepared according to published protocols.^53,62^ Red blood cells were removed by lysis buffer (00-4300-54, eBioscience). Single-cell suspensions were incubated with flow cytometry antibodies targeting surface markers. For intracellular cytokine staining, the suspensions were stimulated using Leukocyte Activation Cocktail with BD GolgiPlug (423303, Biolegend) for 5 h. Antibodies against CD45-BV510 (103137, Biolegend), CD3-FITC (553061, BD Pharmingen), CD4-BV421 (100543, Biolegend), CD8-PE-Cy7 (100721, Biolegend), IL-17A-PE (506903, Biolegend) and IFN-γ-APC (505809, Biolegend) were used at a 1:200 dilution ratio. Dead cells were excluded from analysis using the Zombie NIR™ Fixable Viability Kit (423106, Biolegend). Samples were analyzed using a BD LSRFortessa X-20 (BD Biosciences) or CytoFLEX (Beckman Coulter). Results were analyzed using FlowJo 10.5.3 software (BD Biosciences).

### Statistical analysis

The data are presented as the mean ± SEM. Prism (GraphPad Software) was used for statistical analysis. Significant differences were evaluated by unpaired Student’s *t* test or two-way ANOVA. Kruskal‒Wallis test and PERMANOVA were used for microbiota α-diversity and β-diversity analysis. Values of *P* ≤ 0.05 were considered statistically significant.

### Supplementary information

Supplemental materials
