# Supplementary material for: Periodontitis exacerbates pulmonary hypertension by promoting IFNγ+ T cell infiltration in mice
Source: Int J Oral Sci. 2024 Mar 28;16:27. doi: 10.1038/s41368-024-00291-2 (PMC10978940; doi:10.1038/s41368-024-00291-2)
Supplement: Supplementary file 1 — Supplemental materials [file 41368_2024_291_MOESM1_ESM.docx]

**Fig S1**


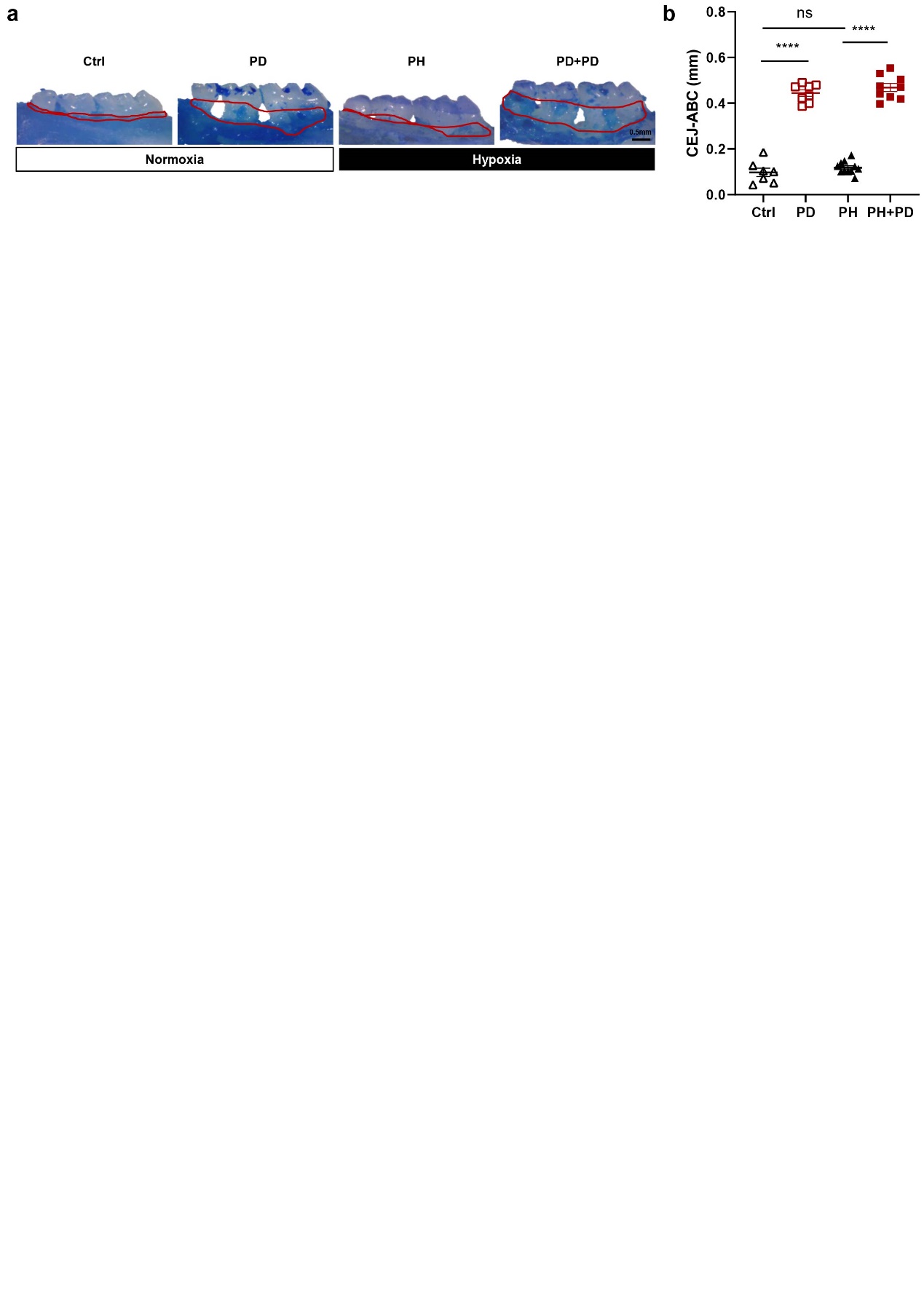


**Fig S1. Effects of PD on alveolar bone loss in mice.** (a) Representative images of mouse maxillae exhibiting alveolar bone loss at the end of the experiment. Red lines depict the space between cementoenamel junction (CEJ) and alveolar bone crest (ABC). (b) Quantification of total CEJ-ABC distance. n=7:8:11:9. Ctrl, nonligatured control. PD, ligature-induced periodontitis with oral infection of PL. PH, pulmonary hypertension. Data are presented as mean ± SEM. Two-way ANOVA (b) was used for statistical analysis. ns, not significant. *****P*<0.0001.

**Fig S2**


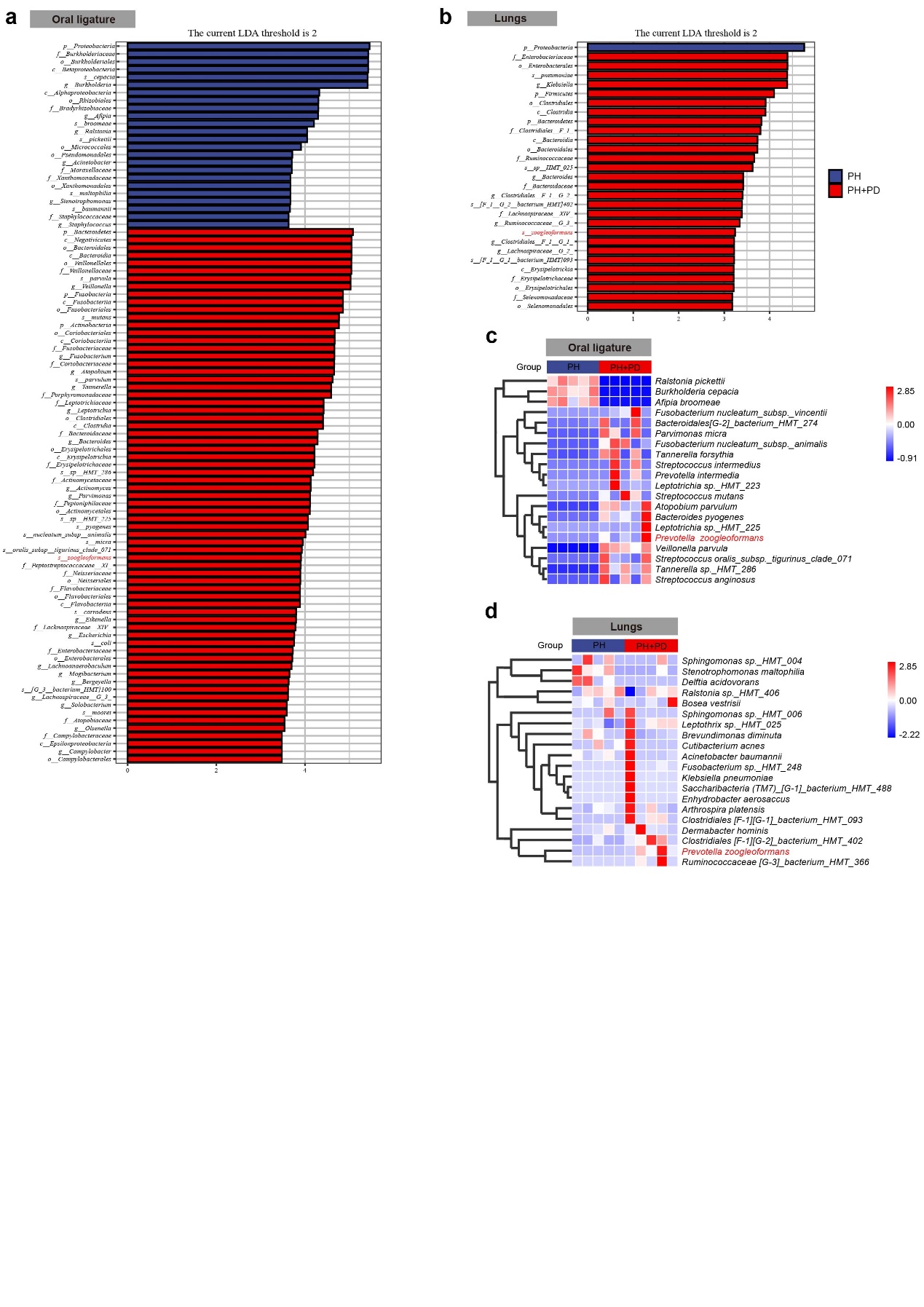


**Fig S2. PD promotes the accumulation of** ***P. zoogleoformans* in the oral cavities and lungs of PH mice.** (a-b) Enriched bacterial taxa in oral ligature (a) and lungs (b) analyzed by linear discriminant analysis effect size (LDA>2). n=5 per group. (c-d) The distribution trend of species abundance in oral ligature (c) and lungs (d). n=5 per group.

**Fig S3**


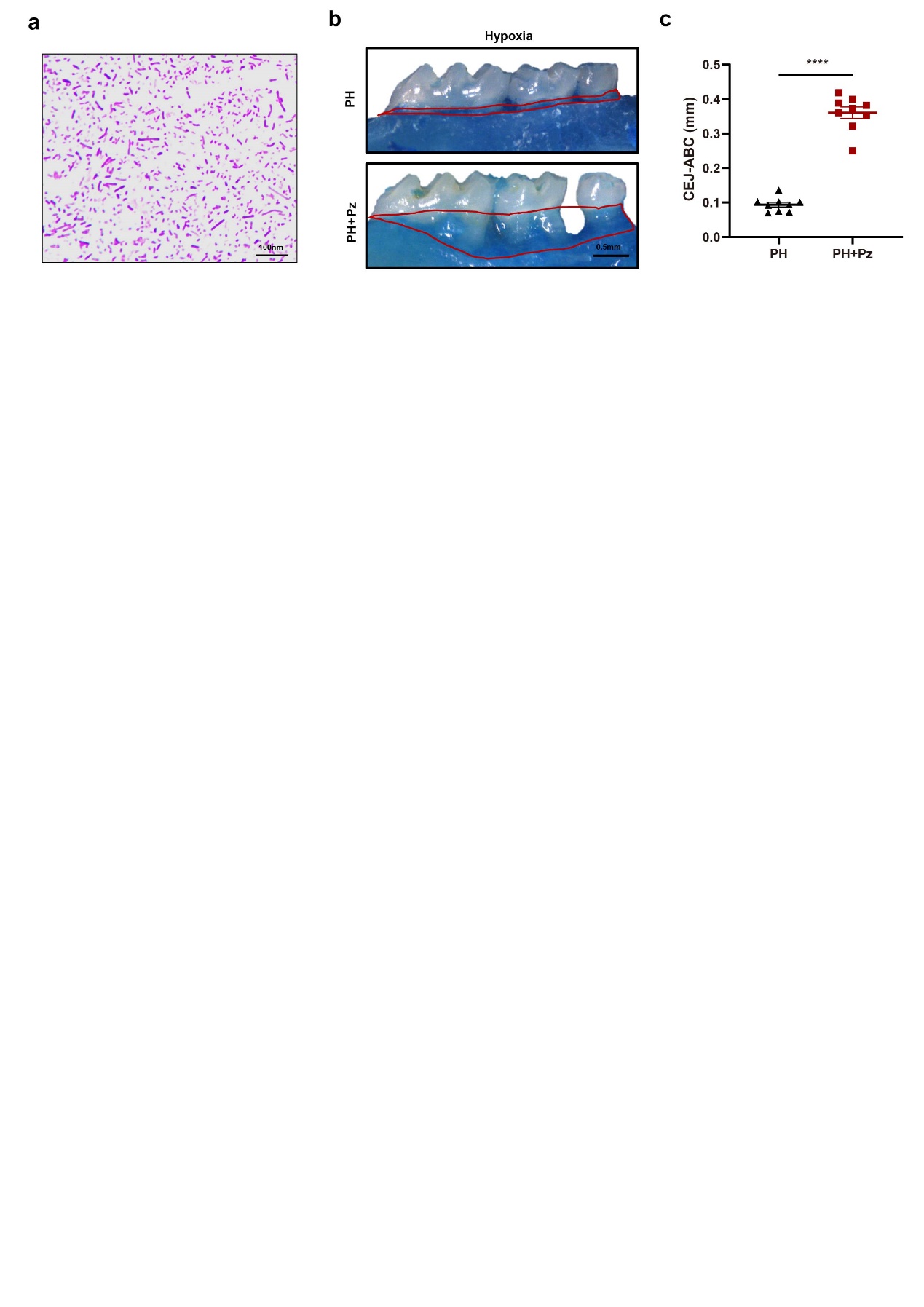


**Fig S3. Effects of *P. zoogleoformans* and PD on alveolar bone loss in mice.** (a) Gram staining of *P. zoogleoformans*. (b) Representative images of mouse maxillae exhibiting alveolar bone loss at the end of the experiment. Red lines depict the space between CEJ and ABC. (c) Quantification of total CEJ-ABC distance. n=9:9. Pz, ligature-induced periodontitis with oral infection of *P. zoogleoformans.* Data are presented as mean ± SEM. Student’s t test (c) was used for statistical analysis. *****P*<0.0001.

**Fig S4**


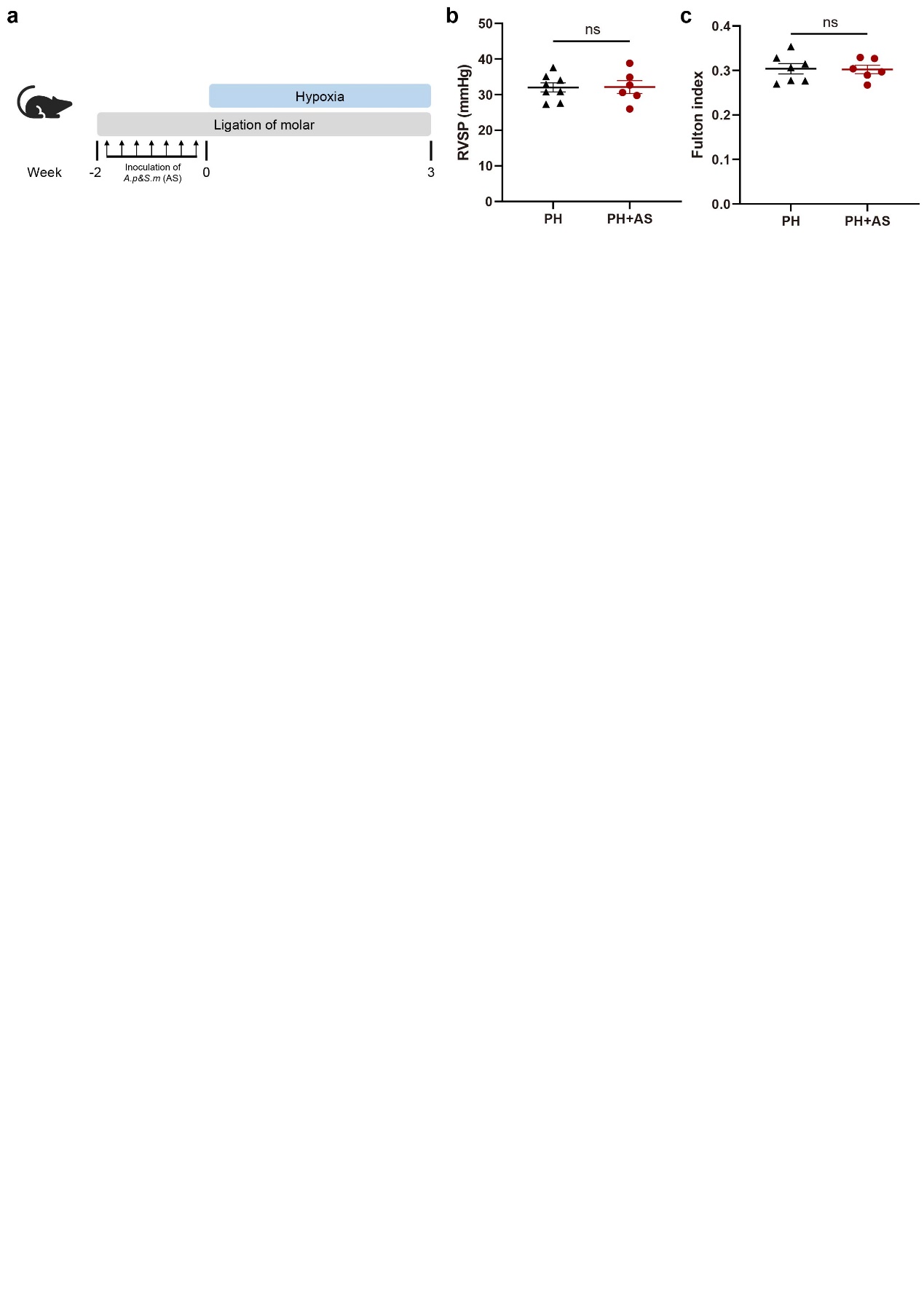


**Fig S4. Oral administration of *A. parvulum* and *S. mutans* mixture (AS) does not exacerbate PH in mice.** (a) Schematic illustration of the experimental design. (b) RVSP and (c) Fulton index determined by the ratio of (RV/(LV + S)). n=7:6 for RVSP, n=8:6 for Fulton index. AS, ligature-induced periodontitis with oral infection of *A. parvulum* and *S. mutans.* Data are presented as mean ± SEM. Student’s *t* test (b, c) was used for statistical analysis. ns, not significant.

**Fig S5**


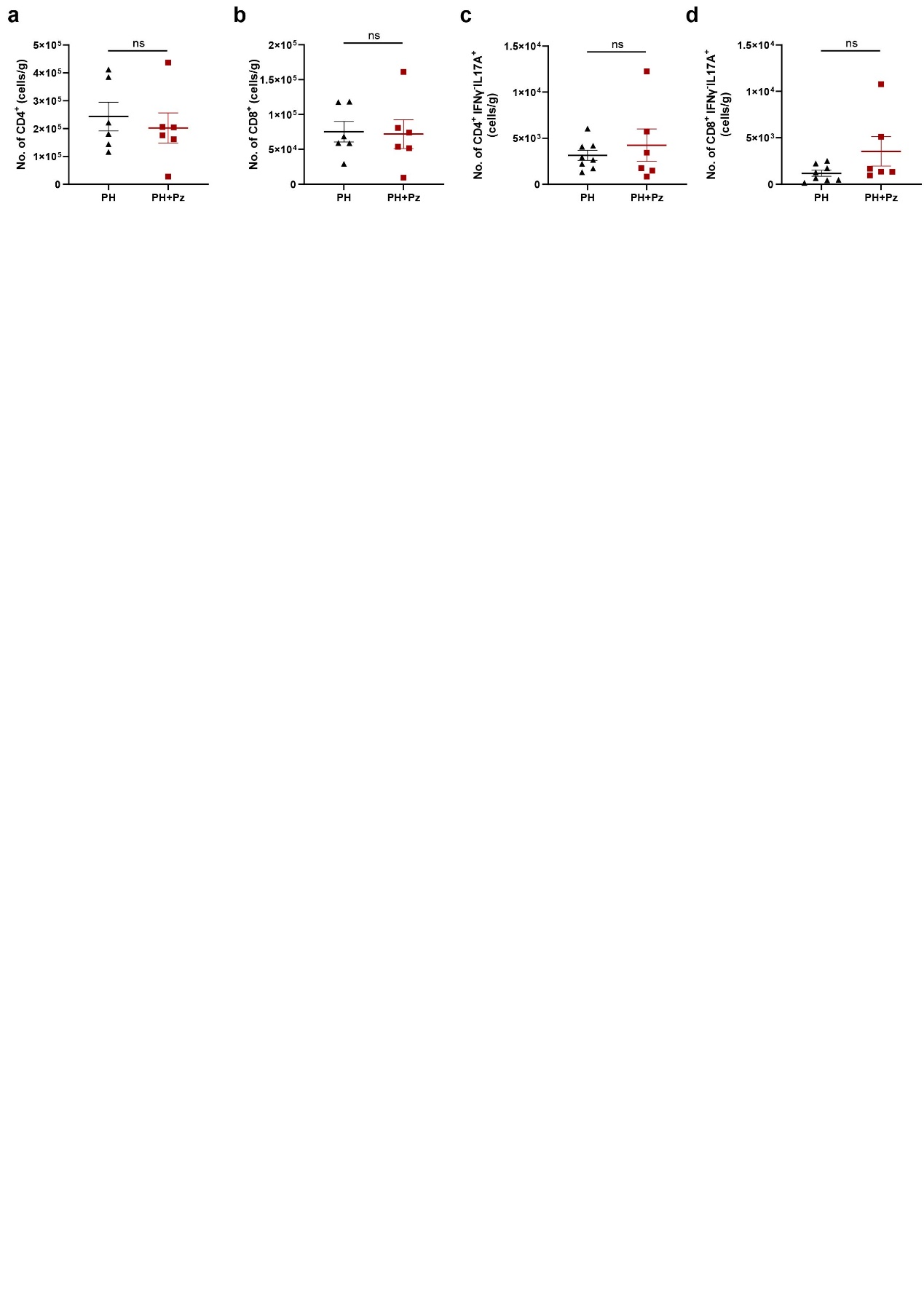


**Fig S5. *P. zoogleoformans* does not affect the number of interleukin-17A positive (IL17A^+^) T cells in lungs of PH mice.**

(a-b) Quantifications of the number of CD4^+^ T cells (a) and CD8^+^ T cells (b) in lungs. n=6:6. (c-d) Quantifications of the number of CD4^+^IL17A^+^ T cells (c) and CD8^+^IL17A^+^ T cells (d) in lungs. n=8:6. Data are presented as mean ± SEM. Student’s *t* test (a, b, c, d) was used for statistical analysis. ns, not significant.

**Fig S6**


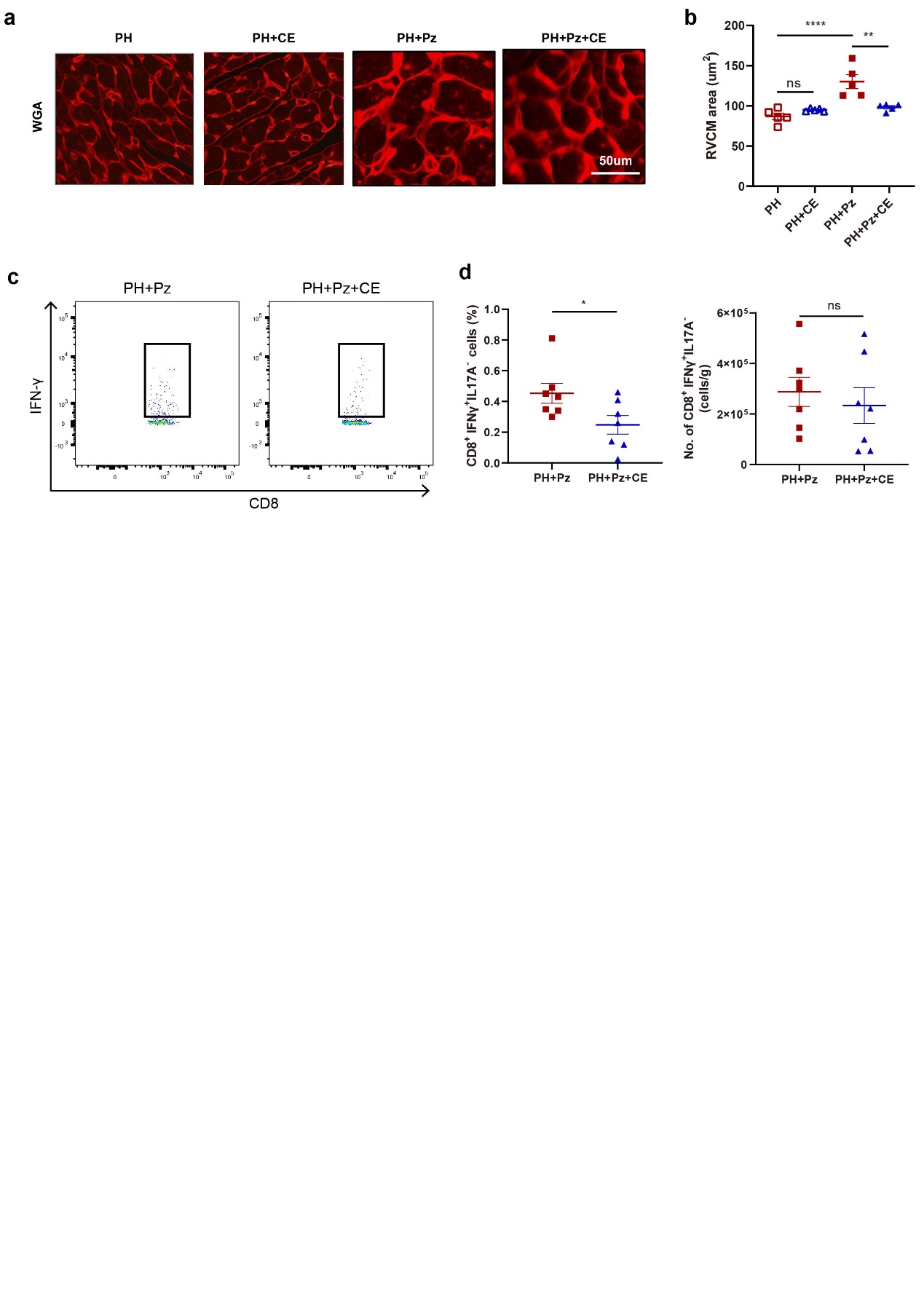
**Fig S6. Surgical excision of cLNs does not affect the number of CD8^+^IFNγ^+^ T cells in lungs of mice with PH and Pz.**

(a) Representative WGA staining of mouse RV sections. (b) Quantification of RVCM area based on WGA staining. n=5: 5:5:5. CE, cLN excision. Pz, ligature-induced periodontitis with oral infection of *P. zoogleoformans.* (c-d) Quantification of the percentage of CD45^+^ cells and number of CD8^+^IFNγ^+^ T cells in mouse lungs. n=8:7. Data are presented as mean ± SEM. Student’s *t* test (b) and Two-way ANOVA (d) were used for statistical analysis. ns, not significant. **P*<0.05.***P*<0.01. *****P*<0.0001.
